# Identifying Practical Solutions to Meet America’s Fiber Needs: Proceedings from the Food & Fiber Summit

**DOI:** 10.3390/nu6072540

**Published:** 2014-07-08

**Authors:** Amy R. Mobley, Julie Miller Jones, Judith Rodriguez, Joanne Slavin, Kathleen M. Zelman

**Affiliations:** 1 Department of Nutritional Sciences, University of Connecticut, Storrs, CT 06269, USA; 2 Department of Family, Consumer and Nutritional Sciences, St. Catherine University, St. Paul, MN 55105, USA; E-Mail: jmjones@stkate.edu; 3 Department of Nutrition & Dietetics, Brooks College of Health, University of North Florida, Jacksonville, FL 32224, USA; E-Mail: jrodrigu@unf.edu; 4 Department of Food Science and Nutrition, University of Minnesota, St. Paul, MN 55108, USA; E-Mail: jslavin@umn.edu; 5 Director of Nutrition, WebMD, Marietta, GA 30062, USA; E-Mail: kzelman@webmd.net

**Keywords:** fiber, whole grains, *Dietary Guidelines for Americans*, nutrient of public health concern

## Abstract

Fiber continues to be singled out as a nutrient of public health concern. Adequate intakes of fiber are associated with reduced risk for cardiovascular disease, cancer, diabetes, certain gastrointestinal disorders and obesity. Despite ongoing efforts to promote adequate fiber through increased vegetable, fruit and whole-grain intakes, average fiber consumption has remained flat at approximately half of the recommended daily amounts. Research indicates that consumers report increasingly attempting to add fiber-containing foods, but there is confusion around fiber in whole grains. The persistent and alarmingly low intakes of fiber prompted the “Food & Fiber Summit,” which assembled nutrition researchers, educators and communicators to explore fiber’s role in public health, current fiber consumption trends and consumer awareness data with the objective of generating opportunities and solutions to help close the fiber gap. The summit outcomes highlight the need to address consumer confusion and improve the understanding of sources of fiber, to recognize the benefits of various types of fibers and to influence future dietary guidance to provide prominence and clarity around meeting daily fiber recommendations through a variety of foods and fiber types. Potential opportunities to increase fiber intake were identified, with emphasis on meal occasions and food categories that offer practical solutions for closing the fiber gap.

## 1. Introduction

Fiber consumption consistently falls short of national recommendations, a trend that has been documented for nearly two decades [1,2]. Less than 5% of most age and gender subgroups have usual intakes of fiber that meet the adequate intake (AI) level of 25 to 38 g/day identified by the Institute of Medicine (IOM) [3,4]. Mean intakes of fiber ranged from 11 to 19 g/day for individuals aged two years and older in 2009–2010, indicating that most Americans need to nearly double their fiber consumption to meet their daily recommendation [4,5]. Fiber was first identified as a nutrient of public health concern in the 2005 *Dietary Guidelines for Americans* (DGA) and was reaffirmed in 2010 [6]. Even with this recognition, fiber intakes have not progressed toward national goals over the past decade, suggesting that fiber will remain a nutrient of public health concern in upcoming dietary guidance. 

Explanations for this fiber intake gap have been explored and proposed [7,8]. Consumer research suggests that many Americans believe they are getting enough daily fiber, yet there is confusion around which foods provide fiber, especially related to whole-grain foods, and how much fiber is needed for good health [9,10]. Current dietary guidance identifies legumes, vegetables, fruits, whole grains and nuts as the best sources of fiber and emphasizes “make half your grains whole” as a key recommendation [6]. Americans may be relying heavily on whole-grain foods as a source of fiber when, in reality, whole grains contain varying amounts of fiber, and many lack the fiber to qualify as a “good source” of fiber (≥3 g/serving) [9,11]. 

To address the ongoing public health concern around the fiber deficit, the Food & Fiber Summit was organized by a steering committee of nutrition researchers and educators. The summit was held on 28 January 2014, in Washington D.C., bringing together leading nutrition and health professionals to explore emerging research on fiber’s role in health, current fiber consumption and consumer awareness trends and ongoing efforts to close the fiber gap. The objectives of the summit were to identify barriers and opportunities for meeting current fiber recommendations and to generate effective strategies and solutions for improving fiber intakes and communicating future dietary guidance. 

## 2. Fiber’s Role in Improving Public Health

A historical perspective of fiber research offered insights into how the understanding of the health benefits of fiber has evolved and the potential role of future fiber research. Prior to 1970, human nutrition advice and knowledge around fiber was based primarily on its role in supporting digestive health and laxation. Subsequently, epidemiological evidence showed that inadequate fiber intake was associated with many chronic diseases seen in the West, but not in populations where fiber intake was high, such as rural Africa [12]. Further, these data suggested that cereal fiber may play a protective role in heart disease, cancer, digestive diseases and obesity, among other conditions [12,13]. Although the proposed mechanisms for fiber’s role in health were simplistic in the early discovery phase, this landmark fiber hypothesis generated interest and prompted further investigation of fiber’s role in health, which continues today. 

Dietary fiber is now recognized as an important component of nutrition and health [14,15,16,17]. The IOM provides daily intake recommendations, and fiber amounts per serving are included on nutrition labels [4]. Based on well-established evidence for an inverse relationship between fiber consumption and the risk for coronary heart disease and certain types of cancer, the U.S. Food and Drug Administration (FDA) approved three health claims for use on food labels stating that increased consumption of fiber can reduce the prevalence of these chronic diseases [18]. Although an AI level has been established for total fiber, the IOM does not classify fiber as an “essential nutrient”, due to the fact that inadequate intakes do not result in clinical or biochemical symptoms of deficiency [4]. However, the short-term effects of an adequate intake of certain dietary fibers, such as bran or psyllium, may include gastrointestinal benefits associated with adding bulk and softness to stools that promote bowel regularity, modulating low-density lipoprotein (LDL) cholesterol and blood pressure, improving blood glucose control in people with diabetes and promoting a feeling of fullness to assist with weight management [19,20,21,22,23]. These short-term benefits can have long-term implications in reducing the risk of gastrointestinal diseases, cardiovascular disease, prediabetes, type 2 diabetes and obesity. While it is well accepted that varying types and components of fibers appear to have different properties that result in different physiological effects, further study is needed to better understand the mechanisms involved. Lattimer and Haub provide a thorough review of current scientific evidence regarding the effects of dietary fiber and its components on metabolic health [16]. Other areas of investigation include fiber’s prebiotic role in supporting a healthy microbiome, with potential benefits related to immunity and inflammation, enhanced bioavailability and uptake of calcium and other minerals, lower risk factors for cardiovascular disease and colon cancer and weight management [24,25,26]. Further research is needed to determine the optimal amounts of fiber, including different types of fiber and its components (naturally occurring and isolated), needed to achieve the benefits that improve health and how to promote a behavior change that results in greater overall intakes of fiber.

Recent research on the economic impact of fiber intake on reducing constipation in the U.S. demonstrated a $12.7 billion direct cost savings in treating functional constipation if adults increased their fiber intake by 9 g/day to recommended levels [27]. This economic model further demonstrated that even if only 50% of the adult population increased dietary fiber intake by 3 g/day, the annual medical cost savings exceeded $2 billion [27].

### Fiber and Whole-Grain Health Benefits

The American Society for Nutrition 2013 position, based on the current state of the science, states that consumption of foods rich in cereal fiber or mixtures of whole grains and bran is modestly associated with a reduced risk of obesity, type 2 diabetes and CVD [22]. Evidence for whole grains alone is limited by varying definitions of whole grains among epidemiological studies of the types and amounts of foods and ingredients included in the whole grain category [22]. Specifically, the inclusion of bran cereals in the whole-grain category used in research studies has been problematic. As a result, the findings observed in the epidemiological research on whole grains can only show a positive association with health outcomes when whole grains and bran are included. Further, recent research reveals that not all whole grains have the same amounts and types of fiber nor exert the same effects [28,29,30]. A review examining the association between whole grains, using the FDA definition for whole grains, and the reduction in the risk for CVD found an association only when the definition of whole grains was broadened to allow studies including isolated bran and germ [31]. Although the scientific evidence continues to evolve regarding the health benefits of whole grains, current evidence suggests that the benefits of consuming whole grains may in large part be due to the fiber content and to the phytochemicals embedded in the bran, along with the fiber. 

It must also be acknowledged that a significant limitation in creating uniform messaging from studies of whole grains and fiber is the lack of whole grain amounts in food composition databases, coupled with the need for a universally-accepted definition of a “whole-grain” food [32,33]. This is particularly problematic in implementing the current dietary recommendation to “make half your grains whole”. Draft guidance on whole grain label statements, issued in 2006 by the FDA, allows food manufacturers to make factual statements about whole grains on product labels, such as “10 grams of whole grains” or “100% whole grain” [34], but there are no rules regarding an amount of whole grain or fiber that must be present. Furthermore, these label statements about whole grains are not consistent with dietary guidance specifying “ounce equivalents” of whole grains, which may also be a factor contributing to the confusion around whole grains [11].

## 3. Current Fiber Consumption in the U.S.

National survey data collected since 2001 indicate that mean daily intakes of fiber have increased approximately 1 g; from 15.1 g in 2001–2002 to 16.2 g in 2009–2010 [35,36]. This may be due to documented increased intakes of whole grain foods for certain populations [37], but also may be attributed in part to the concurrent increase in energy intakes during this time period [35,36]. Males 31–50 year had the highest average intake of fiber at 20 g/day (2009–2010), yet well below the recommended 38 g/day [4,36]. Older females 51–70 year were the group most likely to meet their AI; however, only at the rate of about one in five [36]. Higher fiber intakes (≥80th percentile) were associated with more nutrient- and energy-dense diets, including higher intakes of several micronutrients (potassium, magnesium, iron, copper, folate and vitamin E), greater likelihood of meeting food pattern equivalent recommendations for fruits, vegetables, grains and dairy, as well as higher intakes of macronutrients [36]. 

Grain products and mixed dishes containing grains contribute nearly half of the current intakes of fiber (46%), followed by vegetables (16%), snacks and sweets (13%) and fruits (12%) [36]. This differs from the expected fiber in USDA meal patterns, which are based on average nutrient profiles for food groups, where vegetables should contribute the greatest amount of daily fiber, followed by grains and fruits [38]. It is well established that intakes of vegetables and fruits fall short of recommended amounts [6,39], which also contributes to the fiber intake deficit. Consistently inadequate intakes of fiber have persisted despite a substantial increase in the number of foods promoting whole grain content. According to the Whole Grains Council, the number of products making a “whole-grain” claim increased worldwide by 1.970% between 2000 and 2011, with 3378 new product launches in 2011 [40]. The demand for whole-grain products, rising out of consumer interest from continued emphasis on whole grains in dietary guidance, suggests that consumers may be relying on whole grains with the expectation of increasing their fiber intake. 

## 4. Consumer Confusion on Fiber and Whole Grains

Consumer research indicates that Americans are seeking whole-grain food choices for the purpose of getting more fiber and for the health benefits associated with adequate whole-grain and fiber intakes. The 2013 Food and Health Survey conducted by the International Food Information Council (IFIC) found that more Americans actively made an effort (62%) to get enough fiber and whole grains compared to other food components, including protein, calcium, omega-3 fats, among others [9]. Of the ways Americans are trying to improve their diets, nearly nine out of ten (88%) reported eating more fruits and vegetables, while three out of four (78%) reported eating more foods with whole grains [9]. When questioned about the health benefits associated with whole grains and fiber, respondents were more likely to know that fiber is associated with maintaining a healthy digestive system (85%) and weight management (72%) compared to promoting heart health (52%) or healthy blood sugar (43%) [9]; whereas whole grains were most often associated with heart health (83%), weight management (82%) and a healthy digestive system (81%) and less often with healthy blood sugar (58%) [9]. Whole grains (69%) and fiber (68%) were among the top four food components influencing the decision of what packaged foods to buy [9]. Confusion about where to find fiber and the expectation that foods promoted as whole grains are a good source of fiber may be major contributors to the fiber shortage in Americans’ diets. Whole grains are emphasized in dietary guidance as a source of fiber, yet fiber content varies widely across grains and products promoted as whole grain. Research suggests that consumers consider whole grains and fiber as synonymous with a recent Omnibus Survey revealing that eight out of 10 respondents translated “whole grain” on the product label as a meaning good or excellent source of fiber [10]. As a result, more than half of respondents (56%) believed they were getting enough fiber, and nearly seven out of 10 identified whole grains as one of the best sources of fiber [10].

Marketplace research revealed that many products with whole-grain label statements contain less than a “good source” of fiber, or <3 g fiber per serving. Two recent audits of 212 (2005–2007) and 282 (2009–2011) nationally-distributed ready-to-eat cereal packages in the U.S. with front-of-package whole-grain claims found a wide range of fiber content (0–14 g per serving), with about two-thirds (66%) of whole-grain labeled cereals providing a good to excellent source of fiber in 2009–2011, which represented a 2% increase from 2005 to 2007 [11,41]. Of the 34% of cereals with whole-grain messaging that did not provide at least a good source of fiber in the 2009–2011 audit, nearly two-thirds (61%) provided only 1 g or less of fiber per serving [41]. The lack of regulation governing the use of whole grain labeling has been identified as a factor contributing to the apparent confusion around whole grains and fiber [7,11]. Considering the evidence that consumers are seeking more whole-grain products with an expectation of obtaining fiber, this scenario has the potential to perpetuate the unintended consequence of a continued deficit of fiber, with a possible concurrent increase in energy intakes. Food trends, such as the growing interest in gluten-free, wheat-free and grain-free diets, may further exacerbate the fiber deficit and deserve further investigation [42,43,44].

## 5. Challenges in Communicating Fiber Recommendations

Despite recommendations in dietary guidance and attempts to address the fiber deficit in print, online and social media communications, there has been little to no progress in improving the levels of fiber intake. This may be due to the optimistic perception by some consumers that they are already getting enough fiber, as well as the confusion around good sources of fiber, especially related to whole grains. Regardless, there is a need for renewed attention to fiber in dietary guidance, with the development of consumer-tested coordinated messages and strategies that translate into increased consumption. 

As a nutrient of public health concern in the DGA for the past decade, current recommendations give emphasis to increasing naturally-occurring fiber and are embedded in multi-purpose messages that address foods to increase, specifically in the recommendations to “make half your plate fruits and vegetables” and “make at least half your grains whole grains”. Similarly, MyPlate addresses fiber in second-tier messaging for fruits, vegetables and whole grains. Although this reach may be significant, fiber consumption rates have not responded, suggesting the need for more prominence and clarity in DGA and MyPlate to better identify good sources of fiber and key benefits of increasing fiber intakes that resonate more clearly with consumers.

While national surveys, such as IFIC’s Food and Health Survey, indicate that a significant number of consumers are trying to make higher fiber food purchases [9], a recent online survey of home cooks’ attitudes and behaviors indicate that high in fiber is as important (55%) when picking healthy foods/recipes compared to low in calories (54%) [45]. However, anecdotal evidence from national media outlets suggests that fiber messages in print and online are better received when they focus on a benefit, such as a health outcome or taste. Informal surveys suggest that consumers are not attracted to science-focused messages and need creative and unified messages from trustworthy sources in a platform where they typically obtain information. These surveys suggest that consumers generally are not searching online for “high-fiber” foods, recipes, or lists, but tend to be more interested in specific foods, food categories or occasions (*i.e.*, breakfast) and tangible health benefits. Articles addressing fiber may be more impactful when they “sneak” in fiber messages with recipes and food tips. 

## 6. Fiber Solutions and Future Directions

Conference attendees participated in breakout sessions to discuss key points learned and to identify fiber challenges, opportunities and solutions for consumers, health professionals and future dietary guidance. The results are summarized in Table 1.

A key outcome of the Food & Fiber Summit was that fiber needs a new image. Fiber’s role in health is challenging to communicate due to the multiple types of fiber, varying mechanisms and physiological benefits and the wide range of fiber amounts among foods within food categories [46]. Current messages and strategies to increase fiber intakes have not been successful, suggesting the need for renewed emphasis on education and menu planning to help consumers modify their eating behaviors to choose fiber-containing foods while staying within calorie goals [47]. Innovation to develop good-tasting and affordable foods with more fiber also supports this goal, with the potential to impact public health and lower healthcare costs as fiber intakes increase. 

**Table 1:** Summary of the fiber summit breakout sessions: identifying fiber challenges, opportunities and solutions for consumers, health professionals and future dietary guidance.

Consumers
**Situation and Challenges:** Confusion between fiber and whole grain, the best food sources of fiber and optimal amounts of fiber per day and per meal and snacks. Perceptions that fiber-containing foods do not taste good, cost more and are difficult or time-consuming to prepare. A lack of interest or understanding of fiber; belief that fiber goals are already being met.
**Potential Opportunities and Solutions:** Make messages actionable and provide specific and measurable goals for fiber, e.g., 25 g/day or 8 g/meal, or specific foods with at least 2–3 g/serving. Emphasize that most Americans do not meet daily fiber goals. Address the confusion between fiber and whole grains with messaging that directs consumers to check the fiber content of whole grains. Target messages to focus on the benefits for specific audiences. Focus on quick, easy and affordable tips for getting more fiber from a variety of foods and in meals where fiber intake is the lowest (as a percent of nutrients), such as breakfast and snacks. Raise awareness of fiber through partnerships, such as with celebrities or sports, and consistent messages. Encourage product innovation to increase offerings of fiber-rich food choices.
**Health Professionals**
**Situation and Challenges:** Limited time and competing needs and issues to address with patients and clients. Varying levels of knowledge around fiber types and benefits, including fiber in whole grains. Credible sources and need for user-ready information relevant to the area of practice and client needs (e.g., general health, heart disease, diabetes, gastrointestinal disease, weight management).
**Potential Opportunities and Solutions:** Identify the needs of various health practitioners and leverage trusted sources to share knowledge and education materials. Segment messages and user-ready materials for specific audiences to support behavior change with consistent and easy-to-implement tips. Create a credible presence in social media outlets, online communities and other sources that direct consumers to health professionals and authoritative online sources for fiber guidance. Foster strategic partnerships and initiatives that share consistent messages to help improve credibility and to combat misinformation around grains and carbohydrates.
**Future Dietary Guidance**
**Situation and Challenges:** Fiber messages and food sources are less prominent in key recommendations of the Dietary Guidelines for Americans (DGA), despite fiber being identified as a nutrient of concern. Whole grain messages receive greater attention without specific guidance on choosing whole grains with more fiber, which may be leading to consumer and marketplace confusion about whole grains and fiber.
**Potential Opportunities and Solutions:** Bring fiber to the forefront of DGA recommendations, with greater emphasis on selecting whole-grains based on fiber content. Continue to focus on total diet quality with emphasis on a variety of foods that provide fiber, including vegetables, legumes, fruits, enriched grains, whole grains, bran-based grains and other grain-based foods fortified with fiber. Foods with wide appeal and consumption, such as breakfast cereals and grain-based bars, may offer greater opportunities for increasing fiber intakes. Foster public-private collaboration to bring more fiber to foods that consumers are already eating to address the need for increasing fiber intakes within daily energy goals, e.g., add fiber to whole-grain staples, fast-food sandwich buns, pizza crust.

Consumer confusion around fiber, especially related to whole grains, was identified as a major barrier to meeting fiber recommendations. This confusion may play a role in making some consumers overconfident about the amount of fiber they are getting in their diets. With evidence that over nine out of 10 Americans are not meeting fiber recommendations, future dietary guidance must address the confusion between fiber and whole grains more directly with specific and measurable guidance, such as advice to choose whole grains with at least 3 g of fiber per serving. Public-private partnerships can help communicate a consistent message about fiber and create a fiber commitment among health professionals, the food industry and consumers. 

Science supports the message that various forms and sources of fiber offer health benefits and fit within a healthful eating pattern. Future dietary guidance must continue to emphasize the importance of choosing a variety of foods, including fiber-containing grains, fruits, vegetables and legumes to meet fiber needs within calorie goals and to obtain a range of health benefits. In addition, there must be greater recognition of the role that all forms of fiber can play in closing the fiber gap, including concentrated sources of fiber, such as whole-grain breads and cereals with added fiber. 

Fiber has the potential to play an important role in improving public health; however, the associated health benefits cannot be fully realized until fiber intakes come closer to meeting established recommendations. The Food & Fiber Summit generated several key conclusions, including the need for greater attention to fiber in dietary guidance. Current messages must evolve to effectively address consumer confusion around fiber and whole grains. Segmented and targeted education is needed, with consistent and benefit-driven messages in outlets where consumers look for information from trusted and authoritative sources. Participants of the Food & Fiber Summit identified future opportunities and needs not addressed at the summit, including health professional educational programs that speak more directly and more in depth to fibers’ health benefits and better understanding of consumers’ needs pertaining to fiber and whole grain messaging, particularly in relation to gluten-free diets and growing interest in grain-free eating styles. Overall, there was a consensus that nutrition and health professionals are positioned to create and translate future food and nutrition guidance to help consumers more easily identify, consume and enjoy a variety of fiber-containing foods.
